# Telegenetics for inherited retinal diseases in the COVID-19 environment

**DOI:** 10.1186/s40942-021-00301-z

**Published:** 2021-03-29

**Authors:** Ahmad Al-Moujahed, Aarushi Kumar, Teja Chemudupati, Stephen H. Tsang, Vinit B. Mahajan

**Affiliations:** 1 Molecular Surgery Laboratory, Department of Ophthalmology, Byers Eye Institute, Stanford University, Palo Alto, CA 94304 USA; 2 Department of Ophthalmology, Byers Eye Institute, Stanford University, Palo Alto, CA USA; 3 Department of Ophthalmology, Columbia University, New York, NY USA; 4 Veterans Affairs Palo Alto Health Care System, Palo Alto, CA USA

**Keywords:** Telegenetics, COVID-19, Retina, Inherited retinal diseases, IRDs, Telemedicine

## Abstract

Inherited retinal diseases (IRDs) are visually debilitating conditions that affect families worldwide. They require extensive clinical testing, examination, and patient and family counseling, which are frequently accomplished over single-day extended clinic visits. However, the COVID-19 pandemic has limited the number of patients and staff allowed in clinics, leading to interruptions in care. We therefore developed telehealth management protocols for complete or hybrid virtual visits. The three main components of our telegenetics approach included reviewing the diagnostic tests results remotely, in-person or virtual video visits with a retina specialist, and virtual genetic testing using saliva kits. During the first 5 months of the program, telegenetic care was provided for 80 patients, including 3 international patients, and a spectrum of retinal dystrophies were diagnosed and managed. In conclusion, telegenetic virtual visits ensure continuity of care while reducing patient and provider exposure to SARS-CoV-2 and may continue and expand into other medical genetic conditions long after the pandemic.

Inherited retinal diseases (IRDs) are visually debilitating conditions that affect families worldwide. They require extensive clinical testing, examination, and patient and family counseling, which are frequently accomplished over single-day extended clinic visits [1, 2]. Patients with specific gene mutations may be eligible for gene therapy trials. However, the COVID-19 pandemic has limited the number of patients and staff allowed in clinics, leading to interruptions in care. We therefore developed telehealth management protocols for complete or hybrid virtual visits.

Our telegenetics approach involved three main components (Fig. 1): (1) Results from diagnostic imaging and electrophysiological testing were digitized for remote review by physicians. (2) On a separate in-person or virtual video visit, a retina specialist examined patients or explained clinical test results. Virtual video visits were performed using the MyHealth application, which is Health Insurance Portability and Accountability Act (HIPAA)–compliant, with patients accessing appointments on their mobile phones or tablets. For patients without a video-capable device, a telephone visit was arranged. (3) Genetic testing was entirely virtual. Patients received saliva kits (instead of in-person phlebotomy) by mail and returned samples directly to the laboratory by preposted mail. In-person informed consent with paper forms was converted to telephone consent using online digital forms. Whole exome sequencing reports were delivered by secure download and subsequent review and counseling was completed by video. This dramatically reduced the face-to face time of in-person clinic visits.

**Fig. 1:**
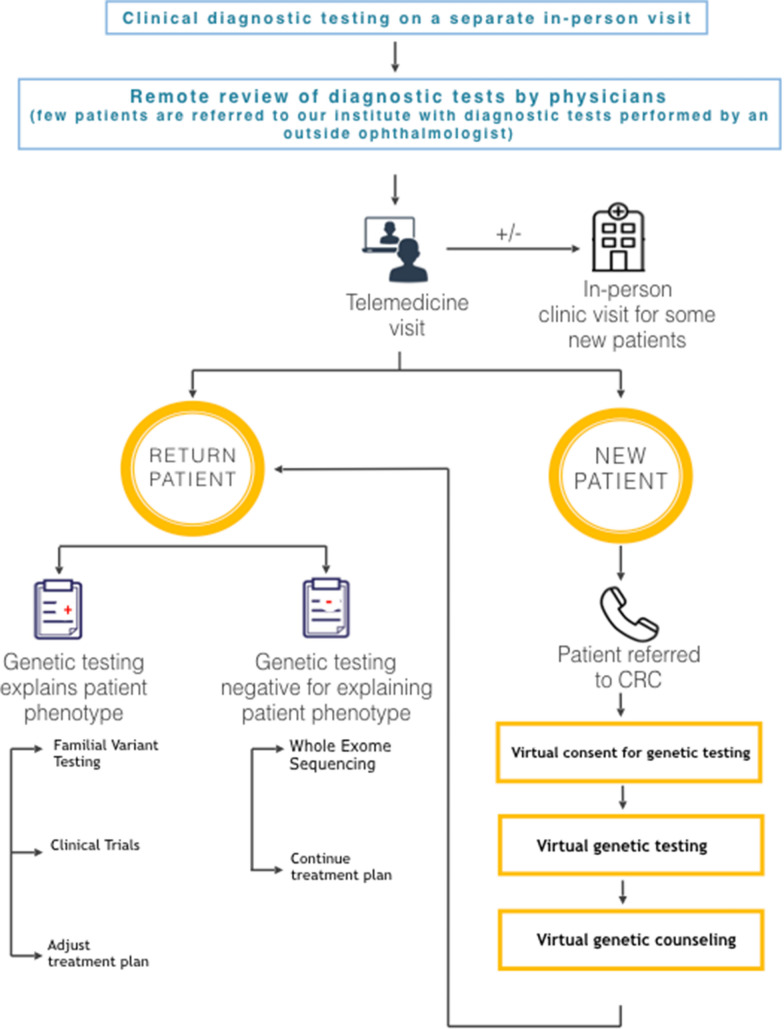
Flowchart that illustrates the process of telegenetics for inherited retinal diseases. CRC: clinical research coordinator

Telegenetic care was provided for 80 patients, including 3 international patients, during the first five months of this program. Nineteen return and 7 new patient evaluations were completely virtual, while 54 were hybrid (39 new and 15 return). The mean age of all patients was 40.8 years (range = 5–74 years) with patients receiving a full virtual evaluation being younger than patients who received a hybrid telegenetics evaluation (mean = 36.8 years, range = 11–62 years and mean = 41.5 years, range = 5–74 years, respectively). A spectrum of retinal dystrophies were diagnosed and pathogenic mutations were detected in several genes, including genes approved for gene therapy or under-investigation in clinical trials like *RPE65*, *CHM*, and *RPGR* [2]. Four patients with cystoid macular edema were successfully treated with topical dorzolamide, and 26 were referred for low-vision rehabilitation.

Previous studies that evaluated cancer telegenetics revealed no difference in patient satisfaction between in-person and virtual care with the latter being more convenient for patients [3–5]. While telegenetic virtual visits do not fully replace in-person visits, they reduce patient and provider exposure to SARS-CoV-2 while ensuring continuity of care. With the recent advancement in telemedicine and virtual communication tools, which accelerated during the COVID-19 pandemic, and their decreased cost compared to the past, we think that implementing a telegenetic program in a setting where IRDs care is available should be feasible with minimal barriers. On the other hand, places where IRDs care is lacking due to poor resources may also have difficulties implementing such a program. However, this kind of program may improve access to remote care for IRD patients living in low-resource settings. With the added efficiency and convenience of virtual patient care, we expect telegenetics will continue and expand into other medical genetic conditions long after the pandemic. Further research is needed to evaluate patient’s satisfaction with this model of eye care.

## Data Availability

Not applicable.

## Abbreviations

IRDs: Inherited retinal diseases
CRC: Clinical research coordinator
